# A phase I/II study of brentuximab vedotin + AVD in paediatric patients with advanced Hodgkin lymphoma

**DOI:** 10.1111/bjh.70207

**Published:** 2025-10-14

**Authors:** Marco A. Salvino, Flavio A. V. Luisi, Mara A. Pianovski, Franca Fagioli, Sidnei Epelman, Luciana B. A. Lima, Vicente O. Filho, Marco Zecca, Claudio Favre, Ryoji Kobayashi, Yuhki Koga, Rizvan Bush, Yulia Sidi, Xiaofei Zhou, Xiang Bai, Frank Campana, Franco Locatelli

**Affiliations:** ^1^ Department of Hematology PPGMS‐UFBA/IDOR‐HSR Salvador Brazil; ^2^ Instituto de Oncologia Pediátrica, GRAACC/UNIFESP São Paulo Brazil; ^3^ Department of Pediatric Oncology Hospital Erastinho, Liga Paranaense de Combate ao Câncer, Jardim das Américas Curitiba Parana Brazil; ^4^ Pediatric Oncology Department Regina Margherita Children's Hospital Turin Italy; ^5^ Department of Public Health and Paediatric Sciences University of Turin Turin Italy; ^6^ Pediatric Oncology Department Hospital Santa Marcelina São Paulo Brazil; ^7^ Hematology, INCA – Instituto Nacional de Câncer Rio de Janeiro Brazil; ^8^ ITACI – Instituto De Tratamento Do Câncer Infantil – Departament De Pediatria – Faculdade De Medicina Da Universidade De São Paulo São Paulo Brazil; ^9^ Oncoematologia Pediatrica, Fondazione IRCCS Policlinico san Matteo Pavia Italy; ^10^ Dipartimento di Oncoematologia Azienda Ospedaliero Universitaria Ospedale Pediatrico Meyer Florence Italy; ^11^ Department of Hematology/Oncology for Children and Adolescents Sapporo Hokuyu Hospital Sapporo Japan; ^12^ Department of Perinatal and Pediatric Medicine Kyushu University Fukuoka Japan; ^13^ Takeda Development Center Americas, Inc. (TDCA) Cambridge Massachusetts USA; ^14^ Department of Pediatric Haematology and Oncology and Cell and Gene Therapy, IRCCS Ospedale Pediatrico Bambino Gesù Catholic University of the Sacred Heart Rome Rome Italy; ^15^ Present address: Merck & Co., Inc. Rahway New Jersey USA

**Keywords:** A + AVD, brentuximab vedotin, Hodgkin lymphoma, paediatric, progression‐free survival

To the Editor,

Significant advances have been made in the treatment of paediatric patients with advanced‐stage classical Hodgkin lymphoma (cHL) since 2010.^1^, ^2^, ^3^ However, current paediatric treatment regimens continue to be based on chemotherapy and radiation therapy, which are associated with potentially serious sequelae, such as secondary malignancies^4^ and infertility.^5^, ^6^ Incorporation of brentuximab vedotin (BV) into standard paediatric multi‐agent regimens has demonstrated increased event‐free survival (EFS) compared with more historical treatment regimens.^1^, ^7^

The Children's Oncology Group recently published a phase III, randomized, open‐label study (AHOD1331; NCT02166463) investigating a modified doxorubicin hydrochloride, bleomycin, vincristine sulphate, etoposide phosphate, prednisone and cyclophosphamide (ABVE‐PC) regimen in which bleomycin was replaced with BV (BV‐AVE‐PC).^1^ BV‐AVE‐PC was associated with a significant improvement in 3‐year EFS compared with ABVE‐PC (92% vs. 83%; hazard ratio 0.41; *p* < 0.001), with no increase in toxicity.^1^ This study led to the Food and Drug Administration approval of BV in combination with AVE‐PC for paediatric patients 2 years of age and older with previously untreated high‐risk cHL.^8^

BV plus doxorubicin, vinblastine and dacarbazine (A + AVD) offers less exposure to cyclophosphamide, etoposide, bleomycin and steroids and their associated long‐term side effects.^9^, ^10^ Therefore, we conducted this phase I/II, open‐label, multi‐agent, multicentre study (NCT02979522) to confirm the recommended dose and assess the safety and efficacy of front‐line A + AVD in paediatric patients with advanced‐stage cHL.

Eligible patients were aged 5 to <18 years and had newly diagnosed, histologically confirmed, advanced (stage III or IV) cHL, with a Lansky Play‐Performance or Karnofsky Performance score of ≥50, and bidimensional measurable disease documented by radiography. Patients were excluded if they had nodular lymphocyte‐predominant HL, known active cerebral/meningeal disease (including signs, symptoms or history of progressive multifocal leucoencephalopathy) or any sensory or motor peripheral neuropathy (PN). The full eligibility criteria are presented in Data S1.

All patients were treated with A + AVD on days 1 and 15 for up to six 28‐day cycles. Phase I aimed to confirm the recommended dose of BV in combination with doxorubicin 25 mg/m^2^, vinblastine 6 mg/m^2^ and dacarbazine 375 mg/m^2^, all administered intravenously on days 1 and 15 according to a modified 3 + 3 design. The paediatric dose selection of 48 mg/m^2^ every 2 weeks (Q2W) was based on extrapolation from adult single‐agent BV exposure data, as detailed in the Supplemental Methods. Definitions of dose‐limiting toxicity (DLT), recommendations for radiation, use of granulocyte colony‐stimulating factor (G‐CSF), assessments, and statistical analyses are available in Data S1.

The trial was performed in accordance with regulatory requirements, the study protocol and good clinical practice guidelines. The protocol was approved by institutional review boards and ethics committees at individual sites. Parents or legal guardians of all patients provided written informed consent. Patients who had reached the age of consent were required to provide a signed informed consent form, while those not of legal age provided assent.

The datasets, including the redacted study protocol, redacted statistical analysis plan and individual participant data supporting the results of the completed study, will be made available after the publication of the final study results within 3 months from initial request to researchers who provide a methodologically sound proposal. The data will be provided after its de‐identification in compliance with applicable privacy laws, data protection and requirements for consent and anonymization.

Phase I primary objectives were to assess safety and tolerability and to identify the recommended dose of BV when combined with AVD for front‐line treatment of advanced‐stage cHL in paediatric patients.

All study patients dosed at the recommended dose were assessed for phase II objectives, which included rates of response per independent review facility (complete remission [CR], partial remission [PR] and overall response rate [ORR]) at end of treatment (EOT); percentage of positron emission tomography (PET)‐negative patients after two cycles; and percentage of patients able to complete six cycles of protocol therapy at the recommended dose.

From 6 September 2017 to 25 September 2019, 63 paediatric patients with previously untreated, newly diagnosed cHL were screened across 14 sites in the United States, Brazil, Japan and Italy. Eight patients were enrolled in phase I, with the DLT determined as BV 48 mg/m^2^ + AVD; 51 patients were enrolled and treated with BV 48 mg/m^2^ + AVD in phase II. All 59 patients from phase I and II completed six cycles of A + AVD. Reasons for screening failure are shown in Figure S1. Patient demographics and baseline disease characteristics are shown in Table S1. Thirty‐two (54%) patients with stage IIIA disease were enrolled.

Responses are summarized in Table 1. All 59 enrolled patients were evaluable for response. At EOT, 52 patients achieved an overall response, resulting in an ORR (CR + PR) of 88% (95% confidence interval [CI], 77–95): 45 (76%) patients had CR (95% CI, 63–86), 7 (12%) had PR (95% CI, 5–23); 4 (7%) were PET‐positive and the remaining 7 (12%) experienced progressive disease (PD) (95% CI, 5–23). PET‐negativity rate after cycle 2 was 90% (95% CI, 79–96) and at EOT was 81% (95% CI, 69–90). Following EOT, 14 patients (24%) received radiotherapy; of these 10 had CR, 1 had PR and 3 had PD. Fourteen (24%) patients had progressed after a median follow‐up of 24.7 months; estimated 24‐month progression‐free survival (PFS) rate was 73% (95% CI, 58–83). Median PFS had not been reached. The estimated 2‐year EFS rate was 72.6%. As of approximately less than 3 years of follow‐up, no enrolled patient had died (estimated 2‐year overall survival rate was 100%).

**TABLE 1:** Summary of response assessments per independent review facility (IRF).

	Phase I (*n* = 8)	Phase II (*n* = 51)	Phase I + II (*n* = 59)
Response at EOT, *n* (%)			
OR	8 (100)	44 (86)	52 (88)
[95% CI]	[63–100]	[74–94]	[77–95]
CR	7 (88)	38 (75)	45 (76)
[95% CI]	[47–100]	[60–86]	[63–86]
PR	1 (13)	6 (12)	7 (12)
[95% CI]	[<1–53]	[4–24]	[5–23]
PD	0	7 (14)	7 (12)
[95% CI]	[N/A]	[6–26]	[5–23]

Abbreviations: CI, confidence interval; CR, complete remission; EOT, end of treatment; N/A, not applicable; OR, overall response; PD, progressive disease; PR, partial remission.

All patients across both phases experienced at least one treatment‐emergent adverse event (TEAE) and most patients (*n* = 51; 86%) had at least one drug‐related grade ≥3 TEAE (Figure S2). The most common any‐grade TEAEs were vomiting (85%), nausea (75%) and neutropenia (58%). The most common grade ≥3 TEAEs were neutropenia (56%), decreased white blood cell count (41%) and decreased neutrophil count (37%) (Figure 1).

**FIGURE 1:**
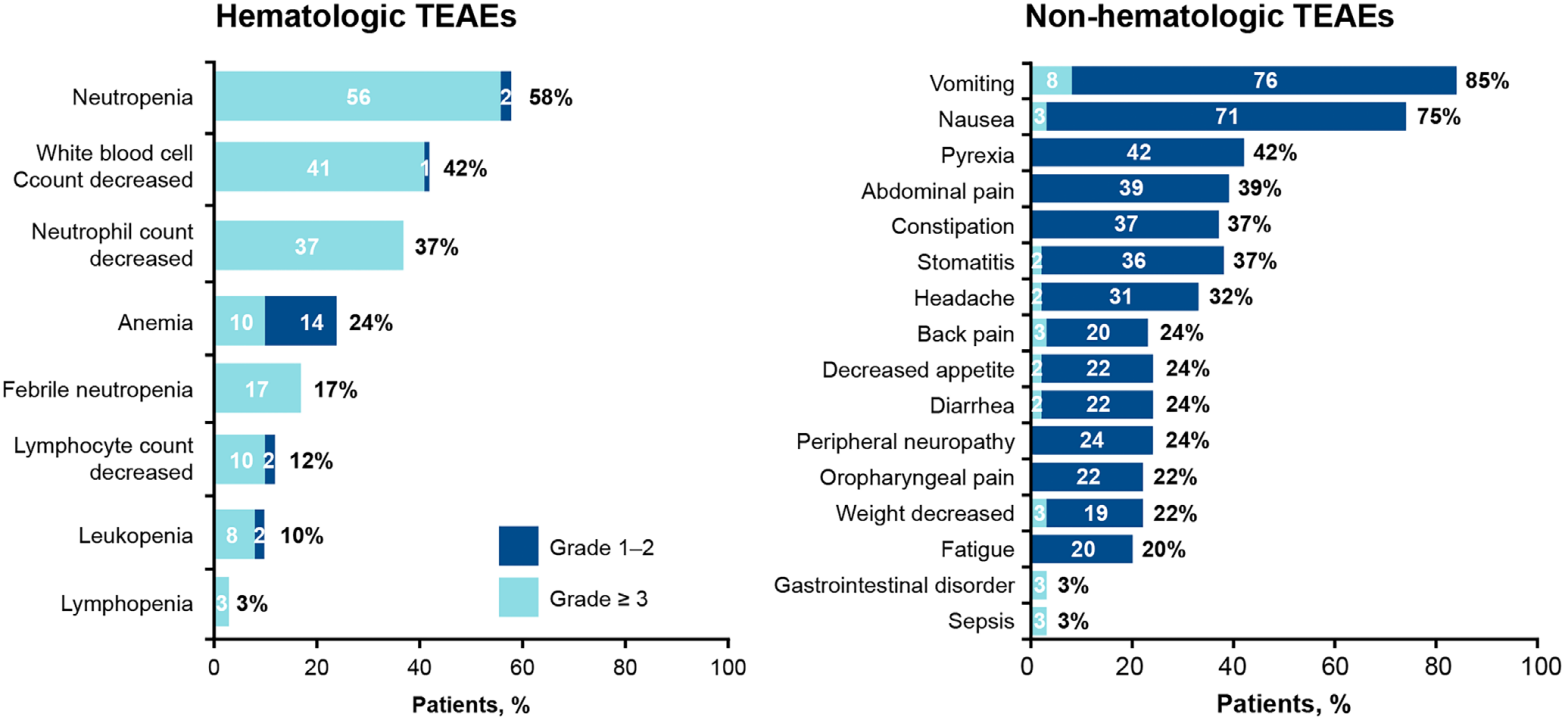
TEAEs in ≥20% of patients at any grade or ≥3% at grade ≥3. TEAE, treatment‐emergent adverse event. Percentages may not sum due to rounding. TEAE, treatment‐emergent adverse event.

In total, 51 (86%) patients had grade ≥3 neutropenia or neutrophil count decreased during the study. Use of G‐CSF at least once (administered according to investigator's judgement) was reported for 37 (63%) patients, with 23 (39%) patients starting first use within cycle 1 (Table S2). Of all nine patients who experienced febrile neutropenia in phase II, only one did not receive G‐CSF (Table S3).

At least one serious adverse event (SAE) occurred in 24 (41%) patients, and SAEs considered to be drug‐related occurred in 19 (32%) patients. Febrile neutropenia was the most common SAE (17% of patients, all considered drug related); SAEs of neutropenia and vomiting were experienced by 5% of patients each. However, no TEAE resulted in premature or permanent discontinuation of treatment, and only three patients required TEAE‐related dose reductions (one patient with grade 4 febrile neutropenia and grade 2 weight decrease, and two patients with grade 2 sensory PN). Neutropenia was the most common TEAE resulting in dose delay (61% of patients required dose delay due to grade 3/4 neutropenia; Table S4), followed by vomiting, decreased white blood cell count, pyrexia and febrile neutropenia. Of those patients experiencing dose delays due to neutropenia or febrile neutropenia, the majority (67% [*n* = 24/36]) had ≤2 dose delays. Two patients experienced TEAEs requiring dose interruptions: grade 3 hypoxia and grade 1 dyspnoea (*n* = 1) and grade 1 vomiting (*n* = 1). In total, 14 of 59 (24%) patients developed treatment‐emergent peripheral neuropathy (PN): 11 (19%) patients experienced grade 1 PN and 3 (5%) experienced grade 2 PN (additional PN results are available in Data S1).

Key serum pharmacokinetic parameters for antibody–drug conjugate (ADC) and monomethyl auristatin E are presented in Table S5. The distribution of ADC clearance, body surface area normalized clearance of ADC and ADC exposure across age groups (<12 years, 12–16 years and >16 years) are displayed in Figure S3.

Additional results (recommended dose determination and treatment exposure, pharmacokinetics and immunogenicity) can be found in Data S1.

In this phase I/II single‐arm, open‐label, multicentre, international study of treatment‐naïve paediatric patients with advanced‐stage cHL, the recommended dosage for BV in combination with AVD was identified as 48 mg/m^2^ Q2W. Treatment with A + AVD, which had not previously been evaluated as a front‐line therapy in paediatric patients with cHL, resulted in an ORR of 88%, PFS of 72.6% and overall survival of 100% after 2 years of follow‐up.

Safety data demonstrated that A + AVD had an acceptable safety profile in patients with advanced‐stage cHL. Consistent with results from the phase III ECHELON‐1 study,^9^ neutropenia was the most common grade ≥3 TEAE. Additionally, although 61% of patients required a dose delay due to neutropenia, no patient discontinued due to neutropenia or any other TEAE. Most non‐haematological TEAEs were grade 1–2, and no deaths were reported up to the current data cut‐off. The incidence of treatment‐emergent PN with A + AVD was lower in the current study (24%) than in ECHELON‐1 (67%).^9^

A + AVD was previously shown to be an effective treatment for an adult population,^9^ and this study demonstrated a 2‐year EFS of 72.6% in paediatric patients. While the recent S1826 trial reported a higher 2‐year EFS of 81% with A + AVD in patients with stage III/IV newly diagnosed cHL,^11^ this rate reflects outcomes in a broader cohort (aged ≥12 years) and does not specifically represent paediatric patients aged 5 to <18 years. It should be noted that, consistent with ECHELON‐1^9^ and with the adult classification of disseminated disease, patients with stage IIIA disease were included in the present trial and accounted for 54% of the enrolled population. We acknowledge that this is inconsistent with other recent paediatric trials, such as AHOD1331,^1^ in which the population included 20% of patients with stage III disease and HLHR13,^3^ which included 25% stage III disease. This could have led to more favourable results in the present trial.

The data from this study support A + AVD as a suitable treatment option for paediatric patients with newly diagnosed, advanced‐stage cHL. Long‐term effects of A + AVD treatment in these patients are being monitored.

## AUTHOR CONTRIBUTIONS

FAVL provided substantial contributions to the conception or design of the work. MAS, FAVL, MAP, FF, SE, LBAL, VOF, MZ, CF, RK, YK, RB, YS, XZ, XB, FC and FL provided substantial contributions to the acquisition, analysis or interpretation of data for the work. All authors were responsible for drafting the work or revising it critically for important intellectual content, providing final approval of the version to be published and agreement to be accountable for all aspects of the work in ensuring that questions related to the accuracy or integrity of any part of the work are appropriately investigated and resolved.

## FUNDING INFORMATION

This work was sponsored by Takeda Development Center Americas, Inc. (TDCA), Cambridge, MA, USA.

## CONFLICT OF INTEREST STATEMENT

MAS reports during the period of the trial consulting or advisory role for Takeda, Novartis, AstraZeneca, Roche, Amgen, AbbVie, Gilead and Johnson & Johnson; serving on a speakers' bureau for Takeda, Novartis, AstraZeneca, Roche, Amgen, AbbVie, Gilead and Johnson & Johnson; reports present employment with AstraZeneca. FAVL reports serving on a speakers' bureau for and receiving research funding from Merck Sharp & Dohme. MAP has received research funding from Millennium Pharmaceuticals. FF has been on an advisory council or committee for Gilead, Iqone (Clingen), Takeda, Bayer, Amgen, Novartis and Eusa Pharma; received research funding from Jazz Pharmaceuticals, Novartis, EUSA Pharma, Gilead Sciences and Takeda; training activities (e.g. ECM, preceptorship) for Amgen, Medac Pharma, Bluebird, Astellas; individual scientific advise for Medac Pharma; and PI or investigator for Eisai, Novartis, Eli Lilly, Takeda, Merck, Incyte, AstraZeneca, Shire, Bellicum, Pfizer, Roche, Alexion, Atara, Allovir, Syndax, Novlmmune, Array, Biopharma Inc., Celgene Corporation and Bristol Myers Squibb. MZ reports a consulting or advisory role for Bluebird Bio, Jazz Pharmaceuticals, Medac and Vertex, serving on a speakers' bureau for Chimerix, providing expert testimony for Novartis as well as receiving travel/accommodations/expenses from Medac. RB is employed by Takeda. YS reports employment and stock and other ownership interests for Takeda and Merck. XZ is employed by Takeda. XB reports employment with Karyopharm Therapeutics, Takeda and Biogen as well as stock and other ownership interests for Summit Therapeutics, Biogen and Titan Pharmaceuticals. FC is employed by and has stock and other ownership interests with Takeda. FL has held a consulting or advisory role for Amgen, Novartis, Bellicum Pharmaceuticals, Neovii and Sanofi and has also served on speaker bureaus for Miltenyi Biotec, Bellicum Pharmaceuticals, Amgen, Medac, Neovii, Gilead Sciences, Takeda, Bluebird Bio, SOBI and Jazz Pharmaceuticals. SE, LBAL, VOF, CF, RK and YK declare no conflict of interests.

## TRIAL REGISTRATION

ClinicalTrials.gov number: NCT02979522.

## Supporting information


**Table S1.** Baseline demographics and disease characteristics.
**Table S2.** Timing of first treatment‐emergent G‐CSF use.
**Table S3.** Treatment‐emergent febrile neutropenia versus G‐CSF use per treatment cycle.
**Table S4.** Neutropenia‐associated dose modifications.*
**Table S5.** Key serum pharmacokinetic parameters following intravenous administration of brentuximab vedotin 48 mg/m^2^ Q2W in cycles 1 and 3 for ADC and MMAE.
**Figure S1.** Patient flow diagram.
**Figure S2.** Safety summary.
**Figure S3.** (A) ADC clearance, (B) BSA‐normalized clearance of ADC and (C) ADC exposure, by age group.
