# Supplementary material for: A phase I/II study of brentuximab vedotin + AVD in paediatric patients with advanced Hodgkin lymphoma
Source: Br J Haematol. 2025 Oct 14;207(6):2583–7. doi: 10.1111/bjh.70207 (PMC12710109; doi:10.1111/bjh.70207)
Supplement: Supplementary file 1 — Table S1. Baseline demographics and disease characteristics. Table S2. Timing of first treatment‐emergent G‐CSF use. Table S3. Treatment‐emergent febrile neutropenia versus G‐CSF use per treatment cycle. Table S4. Neutropenia‐associated dose modifications.* Table S5. Key serum pharmacokinetic parameters following intravenous administration of brentuximab vedotin 48 mg/m2 Q2W in cycles 1 and 3 for ADC and MMAE. Figure S1. Patient flow diagram. Figure S2. Safety summary. Figure S3. (A) ADC clearance, (B) BSA‐normalized clearance of ADC and (C) ADC exposure, by age group. [file BJH-207-2583-s001.docx]

**SUPPLEMENTAL DATA**

**Brentuximab vedotin plus doxorubicin, vinblastine, and dacarbazine in pediatric patients with advanced Hodgkin lymphoma**

METHODS

Inclusion criteria

Each patient must meet all the following inclusion criteria to be enrolled in the study:

1. Male or female patients aged 5 to <18 years.
2. Histologically confirmed CD30+ cHL.
3. Advanced stage, newly diagnosed HL (Stage III and Stage IV disease)
4. Treatment-naïve HL
5. Have performance scores of ≥50 for Lansky Play-Performance or Karnofsky Performance Status
6. Patients must have bidimensional measurable disease as documented by radiographic technique per IWG criteria [1].
7. Female patients who:

- Are surgically sterile, OR
- If they are of childbearing potential, agree to practice 1 highly effective method of
- contraception and 1 additional effective (barrier) method at the same time, from the time of signing the informed consent through 6 months after the last dose of protocol therapy, or
- Agree to practice true abstinence, when this is in line with the preferred and usual lifestyle
- of the subject. (Periodic abstinence [eg, calendar, ovulation, symptothermal, postovulation methods], withdrawal, spermicides only, and lactational amenorrhea are not acceptable methods of contraception. Female and male condoms should not be used together.)

Male patients, even if surgically sterilized (ie, status postvasectomy), who:

- Agree to practice effective barrier contraception during the entire study treatment period and through 6 months after the last dose of protocol therapy, *OR*
- Agree to practice true abstinence, when this is in line with the preferred and usual lifestyle of the subject. (Periodic abstinence [eg, calendar, ovulation, symptothermal, postovulation methods], withdrawal, spermicides only, and lactational amenorrhea are not acceptable methods of contraception. Female and male condoms should not be used together.)

1. Voluntary written consent (and institution-specific assent as appropriate based upon patient comprehension) must be given before the performance of any study-related procedure not part of standard medical care, with the understanding that consent/assent may be withdrawn by the patient or patient guardian at any time without prejudice to future medical care.
2. Suitable venous access for the study-required procedures.
3. Clinical laboratory values within 4 days before the first dose of protocol therapy as follows:

- Absolute neutrophil count ≥1,500/μL unless there is known HL marrow involvement.
- Platelet count ≥75,000/μL unless there is known HL marrow involvement.
- Total bilirubin ≤1.5xthe upper limit of the normal range (ULN) for age or ≤3xULN for
- patients with indirect hyperbilirubinemia due to Gilbert’s syndrome.
- Alanine aminotransferase or aspartate aminotransferase ≤2.5 x ULN for age.
- Creatinine clearance or radioisotope glomerular filtration rate ≥70 mL/min/1.73m^2^ or a serum creatinine based on age/gender as follows:

| Age | Maximum serum creatinine (mg/dL)* | |
| --- | --- | --- |
|  | Male | Female |
| 2 to <6 years | 0.8 | 0.8 |
| 6 to <10 years | 1 | 1 |
| 10 to <13 years | 1.2 | 1.2 |
| 13 to <16 years | 1.5 | 1.4 |
| ≥16 years | 1.7 | 1.4 |

*Derived from the Schwartz formula for estimating, utilizing child length and stature data published by the CDC.

- Hemoglobin ≥8 g/dL (patients may be transfused to meet eligibility criteria).

Exclusion Criteria

1. Nodular lymphocyte predominant HL.
2. Known active cerebral/meningeal disease, including signs or symptoms of progressive

multifocal leukoencephalopathy (PML) or any history of PML.

1. Any sensory or motor peripheral neuropathy.
2. Female patients who are breastfeeding or have a positive serum or urine pregnancy test during the Screening period or a positive serum or urine pregnancy test on Day 1 before the first dose of protocol therapy.
3. Any serious medical or psychiatric illness that could, in the investigator’s or medical monitor’s opinion, potentially interfere with the completion of treatment according to this protocol.
4. Symptomatic neurologic disease compromising normal activities of daily living or requiring medications.
5. Any active systemic viral, bacterial, or fungal infection requiring systemic antibiotics within 2 weeks before the first study protocol therapy.
6. Known hypersensitivity to recombinant proteins, murine proteins, or to any excipient contained in the drug formulation of brentuximab vedotin or any component of AVD.
7. Known human immunodeficiency virus positive.
8. Known hepatitis B surface antigen positive or known or suspected active hepatitis C infection, as determined by hepatitis B DNA or hepatitis C RNA, respectively, in blood.
9. Diagnosed or treated for another malignancy within 3 years before the first dose or previously diagnosed with another malignancy and have any evidence of residual disease. Patients with nonmelanoma skin cancer or carcinoma in situ of any type are not excluded if they have undergone complete resection.
10. Use of any strong or listed moderate cytochrome P450 (CYP) 3A4 inhibitors <2 weeks before the first dose of protocol therapy
11. Any of the following cardiovascular conditions or values within 6 months before the first dose of protocol therapy:

- Shortening fraction of <27% by echocardiogram or, if echocardiogram not feasible,
- ejection fraction of <50% by radionuclide angiogram (RNA or MUGA [multiple-gated
- acquisition scan]).
- New York Heart Association Class III or IV heart failure
- Evidence of current uncontrolled cardiovascular conditions, including cardiac arrhythmias,
- congestive heart failure, angina, or electrocardiographic evidence of acute ischemia or active conduction system abnormalities.

**Rational for dose selection**

At the time of study design and initiation, pediatric dosing guidance for BV had not yet been established. The recommended adult dosage for single-agent BV was 1.8 mg/kg every 3 weeks (Q3W) and the phase III ECHELON-1 trial was investigating first-line BV 1.2 mg/kg Q2W in a combination regimen.^1^ Based on adult exposure data from the 1.8 mg/kg dose, 71.5 mg/m² was found to provide similar exposure in pediatric patients aged 4–17 years. Following extrapolation of single-agent exposure to the dosing in a combination regimen in the first-line setting in which adult BV dosing was 1.2 mg/kg Q2W, the pediatric equivalent was calculated as approximately 48 mg/m² Q2W, with potential de-escalation to 36 mg/m^2^ in case of dose-limiting toxicity (DLT). Pharmacokinetics (PK) was assessed to determine the adequacy of a body surface area normalized dosing regimen.

**DLT definition and observation period**

DLT was defined as a treatment delay of >14 days, or any non-hematologic grade ≥3 toxicity considered by the investigator to be related to brentuximab vedotin (BV), except those occurring in the absence of optimal supportive therapy with duration of >5 consecutive days, and those controllable to grade ≤2 with appropriate treatment (excluding alopecia). The DLT observation period was defined as cycle 1+28 days (from first dose in cycle 1 to day 56). If no or 1 patient experienced a DLT, 48 mg/m^2^ was confirmed as the recommended dose. If >1 patient experienced a DLT, the dose would be reduced to 36 mg/m^2^. If >1 patient receiving 36 mg/m^2^ experienced a DLT, the study would be discontinued. Available pharmacokinetics and safety data were reviewed to guide the final decision on the recommended dose. In phase 1, no granulocyte colony stimulating factor (G-CSF) administration was permitted during the DLT observation period, but was permitted thereafter, per institutional guidelines, for the treatment or prevention of neutropenia.

**Use of G-CSF**

G-CSF use was not permitted during the DLT observation period (cycle 1+28 days: from first dose through study day 56) to avoid risk of confounding observations of potential DLTs related to neutropenia; use of G-CSF prophylaxis was permitted per institutional standards in subsequent cycles, and during phase II. Of note, the requirement to not permit G-CSF use during the DLT observation period was implemented after the first 2 patients had started study treatment and had received G-CSF.

**Recommendations for radiation**

Radiation was administered at the investigator’s discretion and was recommended for patients with positron emission tomography (PET)-positive lesions at the end of cycle 2, with enhanced treatment to areas if lesions remained positive at end of treatment (EOT).

At the end of cycle 2, restaging was performed to assess the status of patients’ disease. For patients who were PET-positive at the end of cycle 2 at the node sites that were PET-positive at diagnosis, involved site radiotherapy was recommended at EOT, and was administered at the investigator’s discretion. Restaging to determine the need for additional radiotherapy was also performed at the EOT disease assessment.

Only residual lymph nodes larger than 1 cm that were still PET-positive at EOT were to be considered for radiotherapy. Additionally, radiotherapy was recommended for patients with extranodal lesions that were PET-positive at the end of cycle 2 or at EOT. For all patients, pericardial and liver irradiation was to be avoided if possible, and radiotherapy was not permitted until patients had completed their EOT disease assessment.

Patients requiring radiation above and below the diaphragm were to be treated with sequential, rather than concurrent fields. The site of bulkiest involvement was to be treated first, and the second field was to be treated with adequate hematologic recovery.

The target volume of irradiation was based on initial nodal and extranodal involvement, as demonstrated by the baseline PET at time of diagnosis. For patients who received radiation at EOT, involved site radiotherapy recommendations were 2100 cGy in 14 fractions of 150 cGy/day, 5 days/week with a boost to sites that were PET+ at the EOT. Treatment was started no later than 4 weeks after completion of the last cycle of chemotherapy, provided that blood count recovery had occurred.

**Assessments**

Response assessments were performed using imaging, including computed tomography (CT), magnetic resonance imaging (MRI), and PET scans, following treatment cycles 2 and 6. After EOT, CT and MRI scans were performed every 24 weeks, up to 2 years. PET-positive patients without progressive disease at EOT had PET scans every 24 weeks, up to 1 year. Response and disease status were evaluated according to International Working Group Revised Criteria for Response Assessment for Malignant Lymphoma by an independent review facility (IRF). PET-positivity was defined as a Deauville score of 4 or 5, while PET-negativity was defined as a Deauville score of ≤3. Survival was assessed until death or up to 2 years after enrollment of the last patient (denoting end of the main study). Eligible patients were invited to participate in an optional 10-year follow-up study.

Toxicity was evaluated using National Cancer Institute Common Terminology Criteria for Adverse Events version 4.03. PK was assessed to determine the adequacy of a body surface area (BSA)-normalized dosing regimen for pediatric patients. Blood samples for measuring antibody-drug conjugate (ADC), monomethyl auristatin E (MMAE), and total antibody (TAb) concentrations were collected in phase I on days 1 and 15 of each cycle and on days 2, 3, 4, 8, 16, 17, 18, and 22 of cycles 1 and 3; in phase II, samples collected between days 16 and 22 of cycles 1 and 3 were omitted. Blood samples for immunogenicity measurements were collected on day 1 of cycles 2, 4, and 6, and at EOT.

**Statistical methods**

Approximately 55 response-evaluable patients treated at the recommended dose were planned to be enrolled in this study. Assuming an overall response rate (ORR) of 90% for BV plus doxorubicin, vinblastine, and dacarbazine (A+AVD) and a two-sided type 1 error of α = 0.2, this would give approximately 78% power to show an ORR of >80%. A final analysis was performed when the last patient enrolled had completed 2 years on study. Analyses were primarily descriptive. Confidence intervals (CIs) for ORR, complete response (CR), and PET-negativity were constructed using the Clopper-Pearson method. Overall survival, progression-free survival, and duration of response were estimated using the Kaplan–Meier method, with corresponding CIs estimated using log-log approach and median follow-up times estimated using a reverse Kaplan–Meier method. Relative dose intensity, expressed as percentage of intended BV dosage, was defined as 100 x (total dose received/total dose intended). Total dose intended was the summation of the intended doses in all treatment cycles; intended dose was the prescribed dose level at cycle 1 day 1 multiplied by actual BSA (m^2^) at each dosing visit. BV ADC, MMAE, and TAb concentration-time data were analyzed by noncompartmental analysis using Phoenix WinNonlin (Version 8.2, Certara, Princeton, NJ). All statistical analyses were performed using SAS 9.4.

**Populations for analysis**

**Safety population**

Patients who received at least one dose of any drug in the A+AVD regimen.

**Response-evaluable population (IRF and investigator)**

Patients who received at least one dose of A+AVD, had measurable disease at baseline, and at least one post-baseline IRF and/or investigator disease assessment.

**Pharmacokinetic population**

Patients with sufficient data to enable calculation of at least one PK parameter.

**DLT-evaluable population**

Patients in the phase I study who received at least one dose of protocol therapy and experienced a DLT during the DLT observation period (cycle 1 day 1 through day 56), and patients who received all planned doses of protocol therapy in cycle 1 and completed all relevant study procedures/assessments during the DLT observation period without a DLT. Patients who were treated in phase I at the dose which was determined to be the recommended dose for phase II were included in the overall response-evaluable patient population.

**Immunogenicity population**

Patients who received at least one dose of study drug and had the baseline immunogenicity sample and at least one post-baseline immunogenicity sample assessment.

**Futility analysis**

An interim analysis (IA) for futility, with a boundary set to 80%, was performed after 25 patients had completed 6 cycles of study treatment and had their EOT response assessment. Therefore, if the upper bound of the 80% CI was below 80%, this would indicate A+AVD had an inferior response (ORR) to standard care therapies that have ORR rates of between 80% and 90%. A conventional 95% CI was also used for the IA. The results of the IA demonstrated that the upper bounds of both CIs did not cross the predefined futility boundary of 80% and therefore no futility was inferred.

**IRF qualifications**

*Appropriate qualifications*

Reviewers qualified by experience, medical knowledge, degrees and medical training, as documented on their curriculum vitae. In addition, new physicians at the IRF underwent a certification process before they were assigned to studies. The certification process provided physicians with training on the process of reviewing images in the blinded independent review setting. New physicians had to complete qualifying training requirements before they became certified IRF readers. Once completed, the readers were certified at the IRF. After certification, they were assigned to studies based on their sub-specialty experience.

*Description of Reviewer Training*

New readers at the IRF were trained according to a new physician training Standard Operating Procedure (SOP). Each independent reviewer assigned to read the protocol for this study received Charter training according to the IRF SOPs.

**RESULTS**

**Recommended dose determination and treatment exposure**

A total of 8 patients were enrolled in phase I and treated in the initial dose cohort of BV 48 mg/m^2^ +AVD. Two patients were not DLT-evaluable per protocol, because they received G-CSF during the DLT observation period. DLTs were evaluated in the remaining 6 patients. No patient experienced toxicities meeting DLT criteria, and no maximum tolerated dose was established. BV 48 mg/m^2^ was, therefore, determined to be the recommended dose in combination with AVD. The mean relative dose intensity of BV was 99.4% (standard deviation 2.4%), and median (range) duration of treatment was 25.3 (23.7–31.1) weeks.

**Peripheral neuropathy**

PN had completely resolved in 10 of 14 patients (71%) at EOT, and 13 of 14 (93%) at last follow-up. Median time to resolution was 1.6 weeks (range, 0.3–100.3). At last analysis, 1 patient had ongoing PN (grade 1 at onset and last follow-up).

**Use of G-CSF**

The median duration of first G-CSF use at any time from cycle 1 to 30 days after cycle 6 was 3 days (range 1–129). The timing of first use and median duration of G-CSF per cycle are shown in Supplemental Table 2.

**Pharmacokinetics**

ADC and TAb peak serum concentrations occurred approximately at the end of infusion. The median time of first occurrence of maximum observed concentration of MMAE occurred in plasma approximately 2 days post dose (Supplemental Table 4). Both ADC and TAb serum concentrations declined in a multiexponential manner, with a terminal disposition phase half-life (t_1/2z_) of approximately 4 days. Accumulation of ADC in serum was minimal at approximately 1.3-fold with every 2 weeks (Q2W) dosing at 48 mg/m^2^ of BV. MMAE plasma concentrations declined log linearly with a mean t_1/2z_ of approximately 2 days. No accumulation of MMAE in plasma was observed following repeated doses of BV at 48 mg/m^2^ Q2W. The distribution of ADC clearance indicated a trend in relation to age (Supplemental Figure 3A). After normalization for BSA, there were no readily apparent differences in ADC clearance among patients across age groups (<12 years, 12–16 years, and >16 years; Supplemental Figure 3B). ADC exposures were comparable across age groups (Supplemental Figure 3C).

**Immunogenicity**

Four patients (7%) were transiently ADA-positive post-baseline, all had low (≤25) ADA titers. One of these patients was ADA positive at baseline; however, the titer did not increase after study drug administration, indicating pre-existing ADA. The other 3 developed ADAs after administration of BV. The ORR in this small group of ADA-positive patients was 100% (including 75% with CR). ADAs were not associated with development of infusion-related reactions.

**Supplementary references**

1. Connors JM, Jurczak W, Straus DJ, et al. Brentuximab Vedotin with Chemotherapy for Stage III or IV Hodgkin's Lymphoma. N Engl J Med. Jan 25 2018;378(4):331-344. doi:10.1056/NEJMoa1708984

**Supplemental Table 1. Baseline demographics and disease characteristics**

| **Parameter** | **Phase I  (*n* = 8)** | **Phase II**  **(*n* = 51)** | **Phase I+II  (*n* = 59)** |
| --- | --- | --- | --- |
| Sex, *n* (%)  Male  Female | 4 (50)  4 (50) | 27 (53)  24 (47) | 31 (53)  28 (47) |
| Median age, years (range) | 13 (6–17) | 14 (6–17) | 14 (6–17) |
| Race, *n* (%)  White  Black or African American  Asian  Other  Not reported | 8 (100)  0  0  0  0 | 26 (51)  12 (24)  3 (6)  9 (18)  1 (2) | 34 (58)  12 (20)  3 (5)  9 (15)  1 (2) |
| Ethnicity, *n* (%)  Hispanic or Latino  Not Hispanic or Latino  Not reported | 2 (25)  6 (75)  0 | 21 (41)  26 (51)  4 (8) | 23 (39)  32 (54)  4 (7) |
| Lansky/Karnofsky performance score, *n* (%)  >90  50–90  Median  [range] | 4 (50)  4 (50)  95.0  [80–100] | 20 (39)  26 (51)  90.0  [70–100] | 24 (41)  30 (51)  90.0  [70–100] |
| Any B symptoms, *n* (%)  Ann Arbor stage lll/lV | 2 (25)  1 (13) / 1 (13) | 21 (41)  11 (22) / 10 (20) | 23 (39)  12 (20) / 11 (19) |
| Ann Arbor stage, *n* (%)  III  IV | 5 (63)  3 (38) | 27 (53)  24 (47) | 32 (54)  27 (46) |
| Bone marrow involvement, *n* (%)  Yes  No | 1 (13)  7 (88) | 8 (16)  43 (84) | 9 (15)  50 (85) |
| Extranodal involvement, *n* (%)  No extranodal sites  1 extranodal site  ≥2 extranodal sites | 4 (50)  0  4 (50) | 20 (39)  12 (24)  19 (37) | 24 (41)  12 (20)  23 (39) |

**Supplemental Table 2. Timing of first treatment-emergent G-CSF use**

|  | **Phase I**  **(*n* = 8)** | **Phase II**  **(*n* = 51)** | **Phase I + II**  **(*n* = 59)** |
| --- | --- | --- | --- |
| **First G-CSF use starts within, n (%)** |  |  |  |
| **Any time from cycle 1 to 30 days after cycle 6 end date** | **5 (63)** | **32 (63)** | **37 (63)** |
| **Cycle 1** | **2 (25)** | **21 (41)** | **23 (39)** |
| **Cycle 2** | **1 (13)** | **7 (14)** | **8 (14)** |
| **Cycle 3** | **1 (13)** | **0** | **1 (2)** |
| **Cycle 4** | **1 (13)** | **1 (2)** | **2 (3)** |
| **Cycle 5** | **0** | **2 (4)** | **2 (3)** |
| **From cycle 6 to 30 days after cycle 6 end date** | **0** | **1 (2)** | **1 (2)** |
| **Duration of first G-CSF use within treatment-emergent period (days),* unless otherwise stated** |  |  |  |
| **Any time from cycle 1 to 30 days after cycle 6 end date, n** | **4** | **29** | **33** |
| **Median (range)** | **1.5 (1–3)** | **3.0 (1–129)** | **3.0 (1–129)** |
| **Cycle 1, n** | **2** | **20** | **22** |
| **Median (range)** | **1.5 (1–2)** | **3.0 (1–129)** | **2.5 (1–129)** |
| **Cycle 2, n** | **1** | **5** | **6** |
| **Median (range)** | **3.0 (3–3)** | **4.0 (1–30)** | **3.5 (1–30)** |
| **Cycle 3, n** | **1** | **0** | **1** |
| **Median (range)** | **1.0 (1–1)** | **N/A** | **1.0 (1–1)** |
| **Cycle 4, n** | **0** | **1** | **1** |
| **Median (range)** | **N/A** | **2.0 (2–2)** | **2.0 (2–2)** |
| **Cycle 5, n** | **0** | **2** | **2** |
| **Median (range)** | **N/A** | **3.5 (3–4)** | **3.5 (3–4)** |
| **From cycle 6 to 30 days after cycle 6 end date, n** | **0** | **1** | **1** |
| **Median (range)** | **N/A** | **7.0 (7–7)** | **7.0 (7–7)** |

*The duration of G-CSF use was not calculated if the end date was missing.

G-CSF, granulocyte-colony stimulating factor; N/A, not applicable.

**Supplemental Table 3. Treatment-emergent febrile neutropenia versus G-CSF use per treatment cycle**

| Febrile neutropenia,  *n* (%) | **Phase I (*n* = 8)** | | **Phase II (*n* = 51)** | |
| --- | --- | --- | --- | --- |
| Cycle | G-CSF | No G-CSF | G-CSF | No G-CSF |
| 1 | 0 | 0 | 3 (6) | 0 |
| 2 | 0 | 1 (13) | 2 (4) | 0 |
| 3 | 0 | 0 | 4 (8) | 0 |
| 4 | 0 | 0 | 3 (6) | 0 |
| 5 | 0 | 0 | 1 (2) | 1 (2) |
| 6 | 0 | 0 | 3 (6) | 0 |

In phase I and II respectively, 1 patient and 9 patients had febrile neutropenia. Patients may have received G-CSF in more than one cycle.

G-CSF, granulocyte-colony stimulating factor.

**Supplemental Table 4. Neutropenia-associated dose modifications***

|  | **Phase I (*n* = 8)** | **Phase II (*n* = 51)** | **Phase I+II (*n* = 59)** |
| --- | --- | --- | --- |
| Any neutropenia event^†^ requiring dose modification, *n* (%) | 7 (88) | 29 (57) | 36 (61) |
| Grade 1–2 | 0 | 0 | 0 |
| Grade 3 | 0 | 7 (14) | 7 (12) |
| Grade 4 | 7 (88) | 22 (43) | 29 (49) |
| Any dose modifications as a result of neutropenia, *n* (%) | 7 (88) | 29 (57) | 36 (61) |
| Dose delayed^‡^ | 7 (88) | 29 (57) | 36 (61) |

*Preferred terms of neutropenia and neutrophil count decreased were counted as neutropenia.

^†^For patients with multiple events, the event with the worst grade was used.

^‡^Dose of study drug (brentuximab vedotin) delayed.

**Supplemental Table 5. Key serum pharmacokinetic parameters following intravenous administration of brentuximab vedotin 48 mg/m^2^ Q2W in cycles 1 and 3 for ADC and MMAE**

|  | **Geometric Mean (CV)** | | |
| --- | --- | --- | --- |
| **ADC** |  |  |  |
| **Parameter, unit** | **Cycle (C)/ Day (D)** | **Brentuximab vedotin 48 mg/m^2^** | **N** |
| C_max_, µg/mL | C1/D1  C1/D15  C3/D1  C3/D15 | 22.5 (22.3)  24.5 (20.0)  26.4 (33.3)  25.3 (19.8) | 57  40  55  32 |
| AUC_0-15D_, d* µg/mL | C1/D1  C1/D15  C3/D1  C3/D15 | 46.7 (30.2)  50.4 (39.4)  61.1 (32.7)  60.3 (20.1) | 57  40  54  30 |
| R_ac_ | C1/D15  C3/D1  C3/D15 | 1.1 (32.2)  1.3 (42.9)  1.3 (29.6) | 39  52  28 |
| AUC_ꝏ_, d* µg/mL  t_1/2z,_ d | C1/D1  C1/D1 | 49.1 (30.1)  3.8 (26.9) | 52  52 |
| CL, L/d CL/BSA, L/d/m^2^  V_z_, L  V_z_/BSA (L/m^2^) | C1/D1  C1/D1  C1/D1  C1/D1 | 1.4 (39.4)  1.0 (30.0)  7.7 (46.7)  5.3 (38.5) | 52  52  52  52 |
| **MMAE** |  |  |  |
| t_max_, h^†^ | C1/D1  C1/D15  C3/D1  C3/D15 | 44.4 (19.7–72.0)  42.9 (20.0–72.0)  45.3 (20.3–71.7)  46.0 (20.0–71.7) | 54  37  53  31 |
| C_max_, ng/mL | C1/D1  C1/D15  C3/D1  C3/D15 | 4.9 (52.4)  2.5 (48.3)  1.6 (47.1)  1.5 (38.3) | 54  37  53  31 |
| AUC_0-15D_, d* ng/mL | C1/D1  C1/D15  C3/D1  C3/D15 | 27.2 (54.8)  15.6 (52.7)  10.1 (49.9)  10.2 (46.1) | 47  31  48  20 |
| AUC_ꝏ_, d* ng/mL | C1/D1 | 27.4 (53.0) | 42 |
| t_1/2z_, d | C1/D1 | 2.1 (17.2) | 42 |

ADC, antibody drug conjugate; AUC, area under the plasma concentration-time curve; AUC_ꝏ_, AUC from time 0 to infinity; AUC_0-15d_, AUC from 0 to 15 days; BSA, body surface area; CL, clearance; CL/BSA, BSA-normalized ADC clearance; C_max_, maximum observed concentration; CV, coefficient of variation; D/d, day; g, grams, h, hour; L, liter; MMAE, monomethyl auristatin E; Q2W, every two weeks; R_ac_, drug accumulation ratio; t_1/2z_, terminal disposition phase half-life; t_max_, time of first occurrence of C_max_; V_z_, apparent volume of distribution during the terminal phase; V_z_/BSA, BSA-normalized apparent volume of distribution during the terminal phase.

^†^Data reported are median (min–max).

**Supplemental Figure 1. Patient flow diagram**


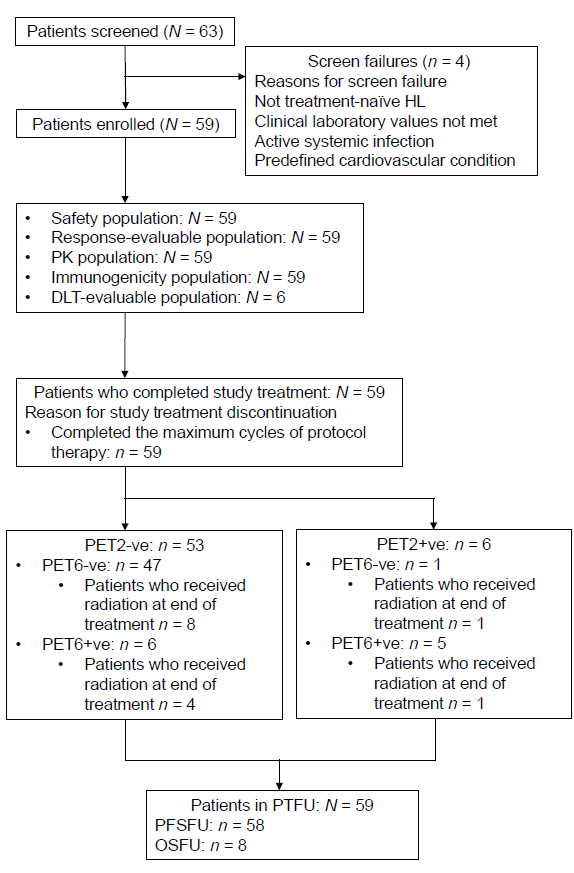


DLT, dose-limiting toxicity; HL, Hodgkin lymphoma; INV, investigator; IRF, independent review facility; OSFU, overall survival follow-up; EOT, end of treatment or after 6 treatment cycles; PFSFU, progression-free survival follow-up; PK, pharmacokinetics; PTFU, post-treatment follow-up.

^*^Per IRF assessment.

^†^PFSFU lasted up to 2 years, with visits every 12 weeks (±1 week) for 12 months and then every 24 weeks (±2 weeks) until either disease progression, death, the start of subsequent anticancer therapy, withdrawal of consent, or end of study.

^‡^After completion of PFSFU, OSFU visits occurred every 24 weeks (±2 weeks) until death or study closure, whichever occurred first, for up to 2 years from enrollment of the final patient.

**Supplemental Figure 2. Safety summary.**

**
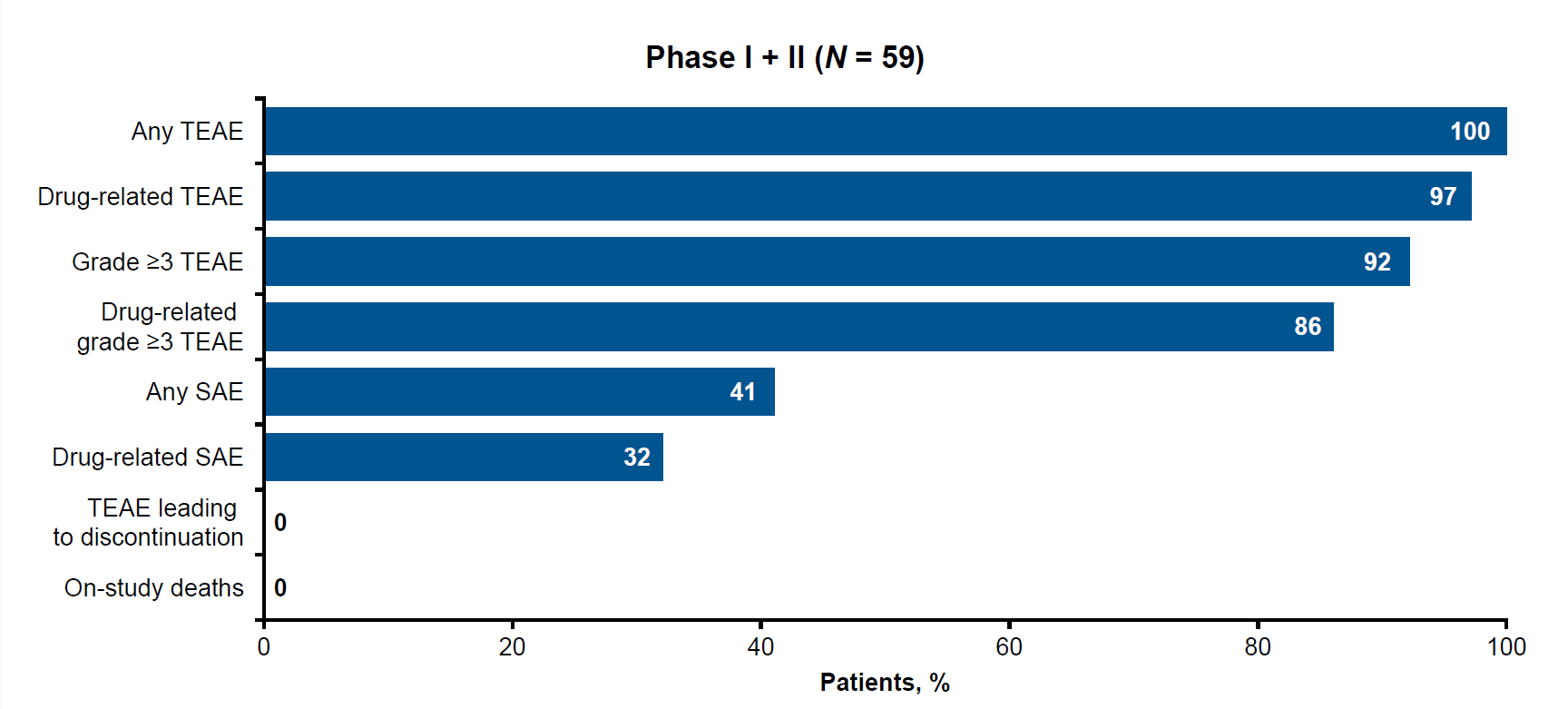
**

SAE, serious adverse event; TEAE, treatment-emergent adverse event.

**Supplemental Figure 3. (A) ADC clearance, (B) BSA-normalized clearance of ADC, and (C) ADC exposure, by age group.**

**
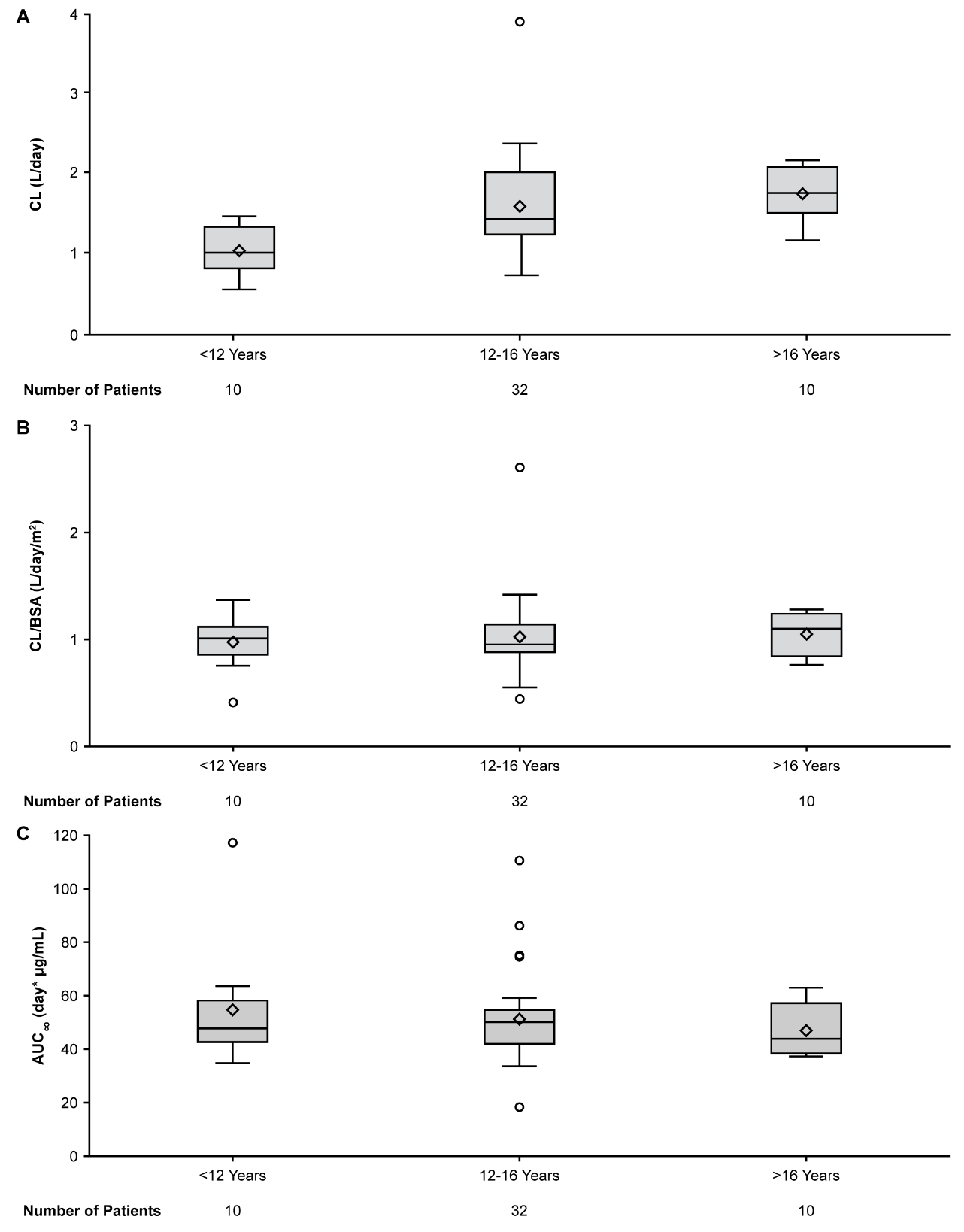
**

ADC, antibody-drug conjugate; AUC_ꝏ_, AUC from time 0 to infinity; BSA, body surface area; CL, clearance; CL/BSA, BSA-normalized ADC clearance; Q1-Q3, quartile1-quartile3.

Horizontal line within box: Median; Diamond: Mean; Box: Q1–Q3. Whiskers end at min/max of quartile -/+ 1.5* interquartile range (IQR), so that all outliers >1.5 times the IQR are individually displayed.
